# Permafrost viremia and immune tweening

**DOI:** 10.6026/97320630019685

**Published:** 2023-06-30

**Authors:** Jaden Penhaskashi, Olivia Sekimoto, Francesco Chiappelli

**Affiliations:** Division of West Valley Dental Implant Center, Encino, CA 91316, USA; Cajulis Pediatric Dentistry, Chula Vista, CA 91914, USA; Dental Group of Sherman Oaks, CA 91403 , USA; Center for the Health Sciences, UCLA, Los Angeles, CA, USA

**Keywords:** Permafrost pathogens, Viremia, Inflammation, Machine Learning, Deep Learning, Deep Ensemble Learning, Molecular Dynamics Simulation, Neuronal Networks, Generative Adversarial Networks, Tracking Responders EXpanding, Immune tweening, TcR[αβ](T cell receptor endowed with the α and β chains), T cell immunity, B cell immunity, CD45R0 (marker of immune cell memory differentiation: ultimate restriction fragment [0] of the common leukocyte antigen, cluster of differentiation [CD]45), CD45RA (marker of naive CD4 and CD8 T cells, first restriction fragment [A] of CD45), TRegs (CD4/8+CD45RA+/R0+FoxP3+), CD279 (programmed cell death marker-1), CD62L (l-selectin, marker of T cells migration), CD25 (α-chain of the interleukin[IL]2 receptor, marker of T cell activation), Tim-3 (T cell immunoglobulin and mucin domain 3), GlycA (glycoprotein acetylation, systemic biomarker of systemic inflammation and autoimmunity), Autologous Immune Enhancement Therapy

## Abstract

The immune system, an exquisitely regulated physiological system, utilizes a wide spectrum of soluble factors and multiple cell populations and subpopulations at diverse states of maturation to monitor and protect the organism against foreign organisms. Immune surveillance is ensured by distinguishing self-antigens from self-associated with non-self (e.g., viral) peptides presented by major histocompatibility complexes (MHC). Pathology is often identified as unregulated inflammatory responses (e.g., cytokine storm), or recognizing self as a non-self entity (i.e., auto-immunity). Artificial intelligence (AI), and in particular specific machine learning (ML) paradigms (e.g., Deep Learning [DL]) proffer powerful algorithms to better understand and more accurately predict immune responses, immune regulation and homeostasis, and immune reactivity to challenges (i.e., immune allostasis) by their intrinsic ability to interpret immune parameters, pathways and events by analyzing large amounts of complex data and drawing predictive inferences (i.e., immune tweening). We propose here that DL models play an increasingly significant role in better defining and characterizing immunological surveillance to ancient and novel virus species released by thawing permafrost.

## Background:

In our previous work [1], we explored the applications and implications of AI in bio-modeling viral immune surveillance. In a simple linear multiple regression model, we posited the desired immune response outcome as Y, the ideal state of immune balance and immune physiological homeostasis following a viral infection. We proposed that Y, the outcome of the complex pattern of physiological, cellular, humoral and molecular immune regulation, can be expressed as the sum of positive and negative immune and psycho-neuroendocrine events. We posited that positive events push immune activation and maturation forward, whereas negative ones impede, blunt or block immunity. In brief, we argued that Y, viz. viral immune surveillance effectiveness, reflects the sum-product of the fine, coordinated and time-regulated interaction of intertwined positive and negative (i.e., immune enhancing and immune suppressive) predictors, which themselves are mediated and regulated by immune cells and soluble factors, and modulated by products of physiological systems distinct from immunity (e.g., hormones, neuropeptides, microRNA's and other molecular factors, etc.) [1]. We stated that gaps in knowledge would soon be filled by AI-assisted immune tweening, which we defined as the AI-aided computerized process of uncovering the initiating substrates, modulating cofactors and end products of individual immune-regulatory sequences that lead to Y. We conceptualized immune tweening as a novel AI-driven process for better understanding the parameters, pathways and events that mediate, modulate and regulate viral immunity [1].

Neural Networks (NNs), the fundamental building blocks of DL, are highly effective when provided with the right information, without requiring constant guidance in the initial stages. The algorithms that characterize DL are typically endowed with multiple layers that progressively identify higher-level features from the raw input [2,4]. We now go beyond our early approach [1] and propose that DL, can, if well trained on AI algorithms focused on biological data, generate models able to perform a variety of complex tasks [2], including immune regulation.

Immune tweening, as originally envisioned [1], necessitates regular temporal updates to establish, for instance, data-driven engines able to indicate the most effective treatments in any patient and virus combination for ensuring anti-viral immune therapy efficacy in general [5]. Nonetheless, these factors may be better defined and characterized by AI protocols developed and tested for a myriad of certain specific viruses and pathogens. Ultimately, DL model variants will be most useful for a broad spectrum of immune therapies, including the design and testing of new generation antibodies [6].

To be clear, AI, and specifically certain models of ML such as DL and its variants, will find a valuable place in the field of viral immunity, particularly in the context of the emerging threat of novel and ancient pathogens released by melting permafrost [7,8]. A more effective and comprehensive understanding of permafrost viral immunity will require improved targeted AI-aided immune tweening by compartmentalizing different components of the immune system as separate variables, and by the underlying assumption that said factors may not be influenced by one another.

## Permafrost viremia and chronic inflammation:

Thawing permafrost releases trapped heavy metals and greenhouse gasses, and a myriad of ancient and novel bacteria, viruses, fungi and parasites. Our immune system is ill-equipped to counter these new challenges, and requires considerable adaptation [7, 8] - a process physiologists term allostasis, the process of transition to and recovery of the equilibrium state of immune homeostasis. In brief, when faced with a foreign pathogen, the immune system launches a multi-faceted immune response to efficiently promote immune allostasis. Multiple bouts of viral infections threaten the wellbeing of individuals and of the general public during allostatic transition to extents that can bring about public health threats not dissimilar to the colliding epidemics the world is now experiencing [9].

Acute viremia generally does not produce a generalized chronic state of inflammation, but chronic viremia, or multiple viremia occurring concurrently can, and does. Inflammation mediates the convergence of tissue-resident and migrating T cells to regulate the transition to immune recovery (i.e., allostasis). Clinically, acute systemic tissue inflammation during viral infection reflects the migration of cytotoxic T lymphocytes (e.g., CD8+CD62L+) and the activation of tissue-resident CD8 T cells (i.e., CD8+CD25+).

Inflammatory responses may become sustained and chronic when steady-state subclinical leukocytic immunity is lost consequential to the concerted immune responses designed to suppress infecting and shedding virus, and suppress virus-infected cells [8,11]. In brief, acute and chronic clinical inflammation can co-exist, and simultaneously alleviate certain aspects, or aggravate other facets of disease. Acute and chronic inflammation can act in clear and distinct manifestations, or in ill-defined intertwined ways, concomitantly or at different time points, depending on the patient and on the viruses involved [10].

In most instances, acute inflammation is a protective response mediated by cell populations of innate immune system (e.g., dendritic cells, myeloid cells) that produce prostaglandins, inflammatory cytokines (e.g., IL1β, IL6) and other pro-inflammatory mediators elicited by injury and infection, which promote immune activation. Chronic inflammation is a sustained damaging response that can lead to cytokine storms, with associated severe pathological damage to a variety of tissues, organs and systems. Chronic inflammation alters the regulation of activation and migration of cytotoxic cells, and impairs viral immunity in part by promoting T cell exhaustion (i.e., CD8+CD279+Tim-3+), and generalized cell-mediated immune suppression. Acute inflammation promotes tissue regeneration and healing and regulates B cell immunity, but chronic inflammation can lead to B cell overdrive and autoimmune reactions, as well as genetic mutations and epigenetic changes in normal tissues [11].

When concomitant infections by novel and ancient viruses released by melting permafrost produce new, and exacerbate existing colliding epidemics and pandemics [9], chronic states of inflammation can be expected to become pervasive. Significant manifestations of immune suppression, rather than activation, with increased number and relative percentage of exhausted CD279+ and Tim-3+ CD8 T cells [12], deregulated inflammatory cytokines [12,13], and impaired B cell responses manifested as autoimmunity [13,14] are expected to occur concomitantly, becoming the norm in the population.

Fundamental and clinical immunologists, alerted to this prospect, must deploy concerted efforts to counter these trends, lest the viremias caused by pathogens in melting permafrost become a gargantuan cross-national public health risk. We argue here that the magnitude of the threat is such that it can only be effectively handled by AI algorithms, such as DL and its variants.

## Deep machine learning for cellular immunity:

Immune surveillance results from complex physiological sets of processes and pathways that intertwine innate and adaptive cellular immunity and that are mediated, modulated and regulated by a myriad of soluble factors that act upon a variety of immune cell populations from lymphoid, myeloid and other origins. Adaptive immunity is mediated by B and T cells [12,14].

T cells drive cellular immunity, and express a vast and diverse repertoire of T cell receptors (e.g., TcR[αβ]). In conjunction with peptide antigen presentation through major histocompatibility complex Class I (MHC-I), CD8+ T cells recognize and are cytotoxic to virus-infected cells. Activated CD8+CD25+ T cells contribute to the adaptive immune repertoire of cytokines and soluble factors that regulate T cell maturation into memory (CD45R0+) cells, T cell exhaustion, and T cell death by apoptosis. Cytokines and factors produced by T cells also regulate B cell maturation and proliferation for the production and release of specific antibody species [12,14].

Proteomics and related techniques have generated a wealth of data on adaptive immune modulation and regulation, and large-scale data sets (i.e., meta-data) have led to the elaboration, testing and evaluation of general ML, and specific DL models for the identification and testing of complex and high-dimensional immune repertoires, including predicting the immunological status of a host to colliding infections with diverse novel and established virus species, and the engineering of relevant immune therapeutics [15], and vaccine development [16]. Having identified these pathways and parameters, immunologists may propose potential treatments such as Autologous Immune Enhancement Therapy (AIET), a process by which native (CD45RA+) T cells are removed from the patient's body where they are manipulated to mature into memory (CD45R0+) T cells by controlled exposure to new and ancient pathogens, and re-administered into the host to boost the immune response [8].

Broadly speaking, the immune repertoires that drive T and B cells maturations are determined largely by sequence-specific transcription factors. The manner in which the DNA sequence of cis-regulatory elements is decoded and orchestrated on the genome largely determines immune cell differentiation lineages. But the specifics of these leverages upon chromatin expression still requires full elucidation. Tailored algorithm architecture, such DL models, elaborated and trained to unravel the hierarchy of transcription factors and their molecular regulators, are key to this knowledge-base.

To be clear, DL modeling is sufficiently powerful and reliable to reveal the regulatory syntax predictive of the full complexity of the differentiation potential of immune cell populations and subpopulations [17]. Maximization of DL predictive potential should, in a relatively close future, generate new molecules with specific predicted biological activity profiles and targeted compound design [18, 19].

## DL models for infectious disease dynamics:

Predicting infectious disease dynamics is a central challenge in disease ecology. DL models can identify the individuals most at risk of exposure to permafrost-released pathogens, provide valuable insights about disease transmission and dynamics, and guide and inform management interventions. DL can incorporate complex nonlinear relationships, with minimal statistical assumptions from ecological data with missing data, and yield enhanced predictive performance, compared to more traditional approaches. DL modeling of immunity will therefore efficiently capture and visualize strong nonlinear patterns and complex interactions between variables in shaping exposure risk from diverse virus infection potentials, and predict not only viral infection risk, but also epidemic ecology severity patterns of morbidity and mortality [20].

In the context of viral immunity, climatic landscapes, and host features are critical, albeit complex, variables in shaping outbreaks of vector-borne diseases. Case in point is the arbovirus bluetongue virus (BTV), a continuously vector-borne pathogen re-emerging among ruminants in the Western Hemisphere, with severe economic implications. ML modeling integrated 23 relevant environmental features and efficiently predicted close to 25,000 outbreaks across 25 Western European countries over a 2-decade time span between 2000 and 2019. The model yielded high predictive performance across all BTV serotypes, and revealed that each of the major BTV serotypes had an outbreak risk profile unique to each geographical location. The algorithm showed strong interactive effects between environmental and host characteristics, and uncovered characteristics of the complex epidemiology of BTV recurrences [21].

Similar AI approaches will elucidate human viral infections, as the ability for viruses to mutate and evade the human immune system remains an obstacle to antiviral and vaccine development. Certain ML models in that regard have used properties such as the influenza hemagglutinin, the HIV-1 envelope glycoprotein, or the spike protein of SARS-CoV2 to predict, with different degrees of statistical success, escape from viral immune surveillance [22]. In brief, AI has been confirmed for its relevance clinically in chemo-informatics, medicinal chemistry and bio-materials development in particular [23,25].

In the specific context of permafrost viral immunity, ML algorithms in general and DL modeling in particular will generate a close approximation of thaw depth and active layer thickness as discrete timelines and seasonal averages. These data will generate important new information on general thaw trends, and estimates of inter-annual changes, and proffer a novel and efficient approach to predict and interpret seasonal permafrost, and the putative release of new and ancient viruses [26].

##  Machine learning and deep learning variants:

In brief, AI has unquestionably advanced infectious-disease surveillance, particularly in the broad context of the pathogens arising from climate change and permafrost melting. However, certain caveats of the widespread utilization of AI in general and DL modeling in particular in healthcare, and specifically in the area of infectious disease remain, including:

1. First, that they impede patient privacy and blunt the patient-centered experience, and

2. Secondly, that they suffer the inherent weakness in sustaining the cross-jurisdictional and cross-functional coordination proffered by National (e.g., CDC) and international organizations (e.g., WHO) that is essential for the collective intelligence required to fight and control novel and emerging infectious diseases, especially in terms of ongoing endemic surveillance for preventing current colliding epidemics and the next pandemic [27].

To revise and expand current AI models, it will be necessary to revisit the original assumptions that have led to their current deployments. Case in point, in its current conceptualization the contemporary scientific model of AI derives from the 1943-design of Turing-complete artificial neurons by US neurophysiologists Warren McCullough (1898-1969) and Walter Pitts (1923-1969) in their notable 1943 [28] and 1948 papers [29]. The seminal work proposed and described the first mathematical model of a fundamental neural network, the McCulloch-Pitts neuron, based on threshold logic algorithms crafted to define and characterize both the fundamental biology of neural networks, and the implication and application of these machine-driven processes, henceforth dubbed 'artificial intelligence' (AI), to analyze and predict neural networks.

Although, in the last eight decades, the McCulloch and Pitts' fundamental paradigm has fast advanced in several primary domains, including learning, reasoning, problem-solving, and decision-making, it has failed to incorporate either of the two issue domains noted by Brownstein and colleagues [27]. That is the primary reason why AI still performs poorly at this time in complex evaluative tasks of moral or ethics, such as what might be found in individualized patient-centered care experiences, or complex multi-National evaluations and assessments. That is not to say that AI cannot handle those tasks; it is simply a reported observation [27] that, at present, it does not. It follows that it is timely and critical that ML models be improved from that perspective.

Indeed as of today, AI, the capability of a computer system to mimic human cognitive functions such as learning and problem-solving, to complex functions and logic to simulate human reasoning, with improved reliability, efficacy and speed, subsumes an array of technologies. Computer software protocols that mimic human cognition to perform complex tasks, learn from them, and repeat them in a replicable manner. It is that very capability that enables the fast, reliable, accurate and replicable performance of a variety of advanced functions along three major domains:

1. artificial narrow intelligence (ANI)

2. artificial general intelligence (AGI) and

3. artificial super intelligence (ASI).

To be clear, the development, testing, evaluation and application of all ML and DL modeling variants must proceed by distinct steps, which include:

1. collect the initial data

2. generate a hypothetical model that fits this preliminary data

3. organize the data to fit the model algorithm

4. run the model - that is to say, 'train' the model

5. evaluate the outcomes of the preliminary runs of the model

6. fine-tune the algorithm model to optimize its fit of the data

7. generate predictions based on the model

8. Expand and refine the model based on new data and findings

ML models, therefore, use algorithms trained on data to perform a variety of complex tasks. ML modeling applications in healthcare in general, and in infectious diseases in particular, are subdivided into three principal domains:

1. Supervised ML, where correct data are inserted in the algorithm to ensure catching and correcting computing errors

2. Unsupervised ML (or reinforcement ML), where correct data are used to confirm and to reinforce the computational algorithm

3. Deep ML, whose algorithms use multiple layers to extract progressively higher-level features from the raw input

To be clear, in the context of clinical immunology, AI has developed with gargantuan leaps forward during the ongoing CoViD-19 pandemic [1, 9, 12, 14, 27]. It now can provide a spectrum of data-based, computer-mediated, distance-processed strategies to monitor the epidemiological impact of infectious diseases, epidemics and pandemics, from tracing trends and predicting peaks of morbidity and mortality, to assessing the effectiveness of novel vaccines and anti-virals, to recording insurance and Medicare coverage and transacting contactless private payments. In brief, AI has strong potential for numerous and undeniably invaluable contributions as a global, interactive, open-source tool to assist health professionals locally and remotely (e.g., tele-medicine, tele-dentistry) in fighting CoViD-19 [30, 31] and other colliding epidemics [9].

The Molecular Dynamics (MD) variant of DL is a relevant computational approach [32]. It has been demonstrated to be reliable to investigate the chemical and structural features of the design of anti-viral drugs and novel vaccines that rely on fractalomic and idiotype/anti-idiotypic interactions [6, 33].

Moreover, the use of unsupervised ML to monitor immune cell subsets in viral and cancer immunity can potentially aid clinical immunologists identify and quantitatively characterize lymphocyte naive (CD45RA+) and regulatory T (FoxP3+)1 cell subpopulations associated with clinical response and immune surveillance, or T cell exhaustion, anergy, apoptosis and disease progression. The ML workflow Tracking Responders EXpanding (T-REX) can, for instance, effectively identify changes in lymphocyte subpopulations based on multi-color flow-cytometry of circulating or tissue-infiltrating immune cells based on phenotype and specific cluster of differentiation expression [34]. T-REX not only identifies biologically significant cells, but also identifies hotspots, and integrates trends that are predictive of disease outcome. T-REX is an efficient ML algorithm that rapidly identifies and characterizes mechanistically significant effector lymphocyte subpopulations, and places emerging diseases into a system's immunopathology context and allostatic recovery trajectory [34].

The protein family of transcription factors endowed with the forehead domain, i,e., Forxhead box, regulate cell proliferation, maturation and longevity. The Fox box family is composed of several classes of proteins, including the FoxP factors that control immune cells pluropotency and maturation. Among these, FoxP3 pertains specifically to T cells, in that it controls and regulates the expression of genes involved in regulating T cell function, and leading to blunting of inflammatory, viral and cancer immunity and auto-immune responses putatively by regulating the expression and translocation of certain suppression-mediating molecules (e.g., extracellular adenosine) into the cytoplasm.

In brief, AI in general, and ML specifically, is a science in its own right. The interpretation of the outcomes generated by algorithms such as T-REX require not only knowledge and training in clinical and fundamental immunology, but, and as importantly, AI expertise. ML specialists in general, and DL experts in particular must be involved alongside allergy and immunology researchers and clinicians in the design, validation, and implementation of AI in immune surveillance to ensure the appropriate development and application of dedicated algorithms, and the correct interpretation and patient-centered utilization of the data generated [35]. This proviso is critical when involving tweening as it is expanded from the domain of, for instance, medical imaging [36] to immune function and phenotypes (i.e., immune tweening, [1]), particularly as it may apply to evolving applications such as immunity to novel and ancient permafrost-released viruses and pathogens [7,8].

## Conclusion:

To explore the potential of ML and DL in immunity, one approach is to create an incomplete simulation of the immune response using Neural Networks (NNs) to predict allostasis. This method requires known inputs and outputs to replicate the simulation for different viruses. Interconnected NNs are populated by raw data, which are then processed through hidden layers and weighted during training to output the desired external data [37]. By such an approach, NNs can be used to describe and predict allostatic responses using data describing the increase or decrease in concentration of certain immune modulators (e.g., microRNA's), neuropeptides (e.g., norepinephrine) and hormones (e.g., steroids), and viral proteomics data (e.g., gene sequence).

To be clear, DL algorithms of viral immunity can be approached and designed in vastly different ways, and with a broad spectrum of predictors, depending on the desired quantifiable input meta-data and output. The choice of variable can range from the very specific, such as the interaction between TcR[αβ]/MHC-I with specific viral coat or envelope proteins [38], to broader immune physiological biomarkers (e.g., glycoprotein acetylation [GlycA]) that alter immune homeostasis or promote allostasis [39,40,41]. Alternate DL algorithms, including Deep Ensemble Learning (DEL), proffer integrated multiple models for more broad-base, precise, accurate and quantifiable predictions [42].

Modeling of immunity in general and permafrost viral immunity in particular cannot be, and must not be a static input-output problem. Rather, it requires constant updating of cellular and humoral immune predictors, molecular effectors, novel and ancient viral species and peptides, and other variables. Computer-based modeling of viral immunity must have the inherent ability to be updated and reinforced based on emerging new data. In that context, deep reinforcement learning (DRL) models [43] are particularly useful in predicting new and emerging epidemics and pandemics.

Specific applications of AI, from DL to DEL and DRL, to permafrost viral immunity, must be trained with fundamental and clinical immunology findings as well as data on existing viruses, and on novel and ancient viral particles released by melting permafrost. As fresh research evolves, the models must be updated and refreshed, and optimized anew [44,45].

Furthermore, the field of AI is fast advancing, and new models and algorithms rapidly developed. Case in point and with respect to gaining a better understanding of the role of interconnected NNs in modeling immune surveillance, generative adversarial networks (GANs) is a novel DL-based approach that employs two interconnected neural networks that compete to generate realistic representations. One network generates synthetic outputs, while the other network discriminates between the generated outputs and real data.

This adversarial process drives the GANs to produce highly convincing and lifelike simulations, proffering an accurate and realistic visualization of biological interactions. GANs may better model immunity, including the emergence of yet unknown viral entities and their interaction with CD8-mediated immune responses. MutaGAN [46] and other GAN-related frameworks [47,48] yield the virtual creation of potentially novel or ancient viruses to (re)appear in the future, and predict their statistical potential to evolve.

In brief, ML can lead to the elucidation of the evolution of ancient hierarchical orthologous viral groups, based on certain patterns of mutations. In certain cases, the evolution of viruses is dominated by loss of distinct features and components of the genome [49], which can determine the epidemic and pandemic potential of new viral species, strains or variants [50]. These techniques (e.g. MutaGAN) are bound to lead to a more nuanced understanding of the general evolution of viruses and the corresponding viral immunity.

Novel and ancient viruses released by melting permafrost may be found to interact with greater fit than anticipated with the human genome. The wide variety of ML models now at our disposal will be most useful in that respect [49, 51]. The oral and oro-pharyngeal cavities, being the first port of entry of most air-borne viruses, as is the case for many fomite-borne viruses as well, can be anticipated to be the initial sites of permafrost viral immunity. Immune tolerance in the oral mucosa is essential to the recognition of native pathogens arising from permafrost thawing and the increase of greenhouse gasses (i.e., CO2 methane gas). It follows that accurate development of ML modeling and immune tweening must consider sites of oral and oral-pharyngeal cavities as a first line of immune defense against ancient pathogens as well as the complexities of permafrost thawing (i.e, active layer thickness, and seasonal averages). Given that the largest extent of the immune response in the oral and oro-pharyngeal cavities is mediated by mucosal immunity, with systemic immunity being relegated mostly to periodontal pockets [52,53, 54], any AI algorithm designed for that purpose need to incorporate mucosal immunity predictors and data, to ensure accurate immune tweening and statistical predictive power.

In conclusion, it is self-evident that, given the vast and expansive potential meta-data that requires analysis and interpretation, increased collaboration between AI experts and clinical and fundamental immunologists is critical. Whereas the immune response must not be oversimplified as an artificial model, careful selection of input types is required to ensure reducing the need for a large quantity of input types and capacity to the essential minimum. By leveraging diverse ML algorithms and models, the most meaningful hyper-parameters that have significant associations will be optimized. That, in turn, will lead to increased predictability and effectiveness, an approach that is already bearing fruit in the discovery of predictive biomarkers for certain cancers [53, 55].
